# Low diet quality increases the genetic risk of high blood cholesterol levels in Asian Indian adults [CURES – 165]

**DOI:** 10.1371/journal.pone.0357182

**Published:** 2026-10-14

**Authors:** Manahil M. Bineid, Dhanasekaran Bodhini, Kandaswamy Ramya, Coimbatore Subramanian Shanthi Rani, Rajendra Pradeepa, Ranjit Mohan Anjana, Venkatesan Radha, Viswanathan Mohan, Karani Santhanakrishnan Vimaleswaran

**Affiliations:** 1 Hugh Sinclair Unit of Human Nutrition, Department of Food and Nutritional Sciences, Institute for Cardiovascular and Metabolic Research (ICMR), University of Reading, Reading, United Kingdom; 2 Department of Food and Nutritional Sciences, College of Agriculture and Food Sciences, King Faisal University, Al-Ahsa, Saudi Arabia; 3 Department of Molecular Genetics, Madras Diabetes Research Foundation, Chennai, India; 4 Department of Clinical Epidemiology, Madras Diabetes Research Foundation, Chennai, India; 5 Department of Research Operations and Diabetes Complications, Madras Diabetes Research Foundation, Chennai, India; 6 Department of Diabetology, Madras Diabetes Research Foundation (ICMR- Collaborating Centre of Excellence) & Dr Mohan’s Diabetes Specialities Centre (IDF Centre of Excellence in Diabetes Care), Chennai, India; 7 The Institute for Food, Nutrition and Health (IFNH), University of Reading, Reading, United Kingdom; Shaheed Rajaei Cardiovascular Medical and Research Center: Rajaie Cardiovascular Medical and Research Center, IRAN, ISLAMIC REPUBLIC OF

## Abstract

**Background:**

Asian Indians face an increased burden of cardiometabolic diseases, yet the interplay between genetic predisposition and diet quality in this population remains underexplored. This study aimed to examine their interaction on cardiometabolic traits in this population.

**Methods:**

We conducted a cross-sectional analysis of 1,020 adults (452 men, 568 women; aged 25–80 years) from the Chennai Urban Rural Epidemiology Study (CURES). A genetic risk score (GRS) of three variants: *TCF7L2* rs7903146, *FTO* rs8050136, and *FTO* rs1588413 was constructed. Diet quality was evaluated using the Indian Diet Quality Score, based on the intake of eight food groups. Associations between the GRS and cardiometabolic traits, as well as the interactions between the GRS and diet quality score, were assessed.

**Results:**

A high GRS was significantly associated with higher fasting plasma glucose (*p* = 0.005) and glycated haemoglobin (*p* = 0.006). Significant interactions between the GRS and the diet quality score were observed on total cholesterol (P_interaction_ = 0.003) and low-density lipoprotein cholesterol (P_interaction_ = 0.005) concentrations, after correction for multiple testing. At the low diet quality score level, individuals with a high GRS had significantly higher total cholesterol and low-density lipoprotein cholesterol concentrations than those with a low GRS (*p* < 0.001, for all comparisons).

**Conclusion:**

This study provides novel evidence for the effect of gene–diet quality interactions on lipid outcomes in Asian Indians. Individuals with higher genetic risk may be more vulnerable to lipid abnormalities when adhering to a low-quality diet, emphasising the value of integrating genetic and dietary data into personalised nutrition strategies for lipid-related cardiometabolic risk prevention.

## Introduction

The global prevalence of cardiometabolic diseases (CMDs), including cardiovascular diseases (CVDs), type 2 diabetes (T2D), obesity and dyslipidaemia, has increased significantly, with low- and middle-income countries the most affected [1–3]. In 2019, Asia accounted for 60% of global CVD-related deaths, highlighting the severity of the crisis in this region [4]. India, the largest country in South Asia, faces a distinctly high burden of CMD, with the increasing prevalence of its risk factors, such as obesity, hypertension and dyslipidaemia [5]. Notably, Asian Indians are more likely to develop metabolic disorders at earlier ages and lower body mass index (BMI) thresholds than Western populations [6,7]. This particular predisposition is attributed to unique metabolic features, lipid profiles [8,9] and a combination of genetic and lifestyle factors [10,11]. India’s ongoing economic and demographic transition has profoundly altered dietary habits, affecting the quality and quantity of the diet consumed. This shift is characterised by excessive calorie intake, predominantly from carbohydrates, as well as inadequate protein, fibre and micronutrient intake; reduced dietary diversity; and an overall decline in diet quality [12,13].

CMD risk is significantly influenced by the interaction between genetic predisposition and lifestyle factors, such as dietary patterns and physical inactivity [14]. Emerging gene–diet interaction studies in South Asian populations underscore the relevance of integrating both genetic risk profiles and lifestyle factors into precision nutrition strategies aimed at reducing susceptibility to metabolic disorders [15]. Genetic association studies have identified a risk relationship between several single-nucleotide polymorphisms (SNPs) and cardiometabolic traits in South Asians [16]. Among these, variations in the fat mass and obesity-associated gene (*FTO)* (Alpha-ketoglutarate-dependent dioxygenase) and the transcription factor 7-like 2 gene (*TCF7L2)* (a subgroup of T-cell-specific transcription factors) have consistently been associated with CMD risk in this population [17–21]. However, alternative genetic approaches that combine several SNPs to build a genetic risk score (GRS) have shown promise, particularly for complex diseases influenced by multiple lifestyle factors [22,23]. Compared to individual SNP analysis, GRS offers a more comprehensive representation of genetic susceptibility and provides enhanced statistical power to uncover gene–lifestyle interactions [24].

Dietary pattern analysis is an increasingly recognised approach for conducting epidemiological research, as it better reflects actual eating behaviours and offers consistent evidence of chronic disease prevention compared to single-nutrient methods [25,26]. Composite diet scores encompass the cumulative impact of multiple dietary components, offering a more comprehensive assessment of diet–disease associations. Only a few studies have applied this approach in the Indian population to identify culturally specific dietary patterns and their association with metabolic risk [27–29]. Although the impact of individual dietary components and macronutrients on genetic associations with cardiometabolic traits has been established in South Asians [15], there is a critical gap in our understanding of how dietary patterns and overall diet quality interact with genetic predisposition to influence CMD risk. To address this gap, this study aims to investigate the understudied complex interaction between genetic risk and diet quality in shaping CMD-related traits in Asian Indians.

## Materials and methods

### Study participants

A total of 1,020 individuals (452 men and 568 women) aged between 25 and 80 years old were recruited randomly from the Chennai Urban Rural Epidemiology Study (CURES) [30]. CURES is a cross-sectional epidemiological study carried out on participants from Chennai City (formerly Madras) in southern India. The methodology of this study has been explained in previous publications [30,31]. In brief, in CURES phase 1, 26,001 adults were recruited between 2001 and 2003 based on a systematic random sampling method. All individuals with diabetes (phase 2, n = 1,382) and one in every ten participants without known diabetes out of the 26,001 phase 1 sample (phase 3, n = 2,207) were invited for additional thorough investigations, constituting the CURES follow-up cohort (n = 3,589) [32]. The current study sample was chosen from the CURES follow-up cohort. For the present study, the data was accessed on 19^th^ of September 2024. All participants provided written informed consent, and the study was granted ethical approval from the Madras Diabetes Research Foundation Institutional Ethics Committee.

### Anthropometric and biochemical measurements

Standardised techniques were used to obtain anthropometric measurements, including height, weight, and waist circumference (WC). The body mass index (BMI) was computed using the following formula: BMI = weight in kilograms (kg) ÷ the square of height in meters (m²). Obesity is defined based on the World Health Organisation Asia-Pacific Guidelines as a BMI of ≥ 25 kg/m^2^ [33]. Biochemical tests were performed utilising the Hitachi-912 Auto Analyser (Hitachi, Mannheim, Germany) with assay kits supplied by Roche Diagnostics (Mannheim, Germany). The following assays were performed: serum total cholesterol (TC) (cholesterol oxidase-phenol-4-amino-antipyrene peroxidase), high-density lipoprotein cholesterol (HDL-C) (direct method; polyethylene glycol-pretreated enzymes) and serum triglycerides (TGs) (glycerol phosphatase oxidase-phenol-4-amino-antipyrene peroxidase). Additionally, low-density lipoprotein cholesterol (LDL-C) was calculated using the Friedewald equation [34]. Fasting plasma glucose (FPG) was estimated using the glucose oxidase-peroxidase method, and glycated haemoglobin (HbA1c) was quantified through high-performance liquid chromatography using a Variant™ analyser (Bio-Rad, Hercules, CA, USA). Diabetes was diagnosed if individuals had a physician-confirmed diagnosis of diabetes (self-reported) or a 2-hour plasma glucose value ≥ 11.1 mmol/L (200 mg/dL) [35]. Individuals with fasting plasma glucose < 5.6 mmol/L (100 mg/dL) and a 2-hour plasma glucose value of 7.8 mmol/L (140 mg/ dL) were classified as having normal glucose tolerance (NGT) [35].

### Dietary assessment

Data on dietary intake were collected using a validated, interviewer-administered semi-quantitative food frequency questionnaire (FFQ) comprising 222 items [36]. Briefly, during a 20- to 30-minute interview, participants were asked to estimate their usual portion sizes and consumption frequencies (daily, weekly, monthly, yearly, or never) for each item listed in the FFQ over the past year. To help with portion estimation, common domestic measures such as bowls, cups, ladles, and spoons were displayed, along with photographic atlases depicting different fruit sizes (small, medium, and large). The recorded dietary data were translated into nutrient intake data using EpiNu® software. The intake of various food groups was also assessed. Details on the development of the FFQ and data on reproducibility and validity have been previously published elsewhere [36].

### Diet quality assessment

Diet quality is scored using the Indian Diet Quality Score (IDQS). The IDQS was previously developed [27] based on food groups identified as positively or negatively associated with T2D incidence. The score was initially applied to the CURES cohort sample. The dietary intake of different food groups was quantified in grams per day. A total of eight food groups, pulses and legumes, milk and its products, fruits and vegetables, white rice, edible fats and oils, added sugar, added salt and poultry and eggs, were included in the score based on their established associations with T2D. To construct the score, food group intakes were adjusted for total energy intake using the residual method. The quintiles for the food groups were calculated, and the mean, minimum and maximum values for each quintile were determined. The eight selected food groups were assigned scores based on their association with T2D. For protective food groups (pulses and legumes, milk and its products, fruits and vegetables), the highest quintile received a score of 10, and the lowest quintile received a score of 0. For harmful food groups, including refined grains and white rice, edible fats and oils, added sugar, added salt, and poultry and eggs, the scoring was reversed: individuals in the highest quintile received a score of 0, while those in the lowest quintile received a score of 10. For the intermediate quintiles (Q2–Q4), proportional scores between 0 and 10 were calculated using the formula described in the original publication [27]. While the type of cooking oil used formed a part of the original score, this analysis revealed a highly skewed distribution for this variable, with sunflower oil being the predominant component. Thus, the cooking oil variable was excluded from the DQS calculation due to its low variability and potential to overlap with the scoring of the edible fats and oils component. A weighting approach was employed to ensure a balanced representation of both protective and harmful food groups in the overall score, adjusting for differences in their quantities to prevent any one group from exerting excessive influence on the final calculations. The score was then divided into tertiles representing levels of diet quality, namely low (< 68.65), medium (68.66–87.87) and high (> 87.88), with higher scores indicating better diet quality. Descriptive statistics for quintiles of food groups are presented in S1 Table.

### Genotyping

The genotyping methodology used has been documented in previous publications [17,21,37]. In brief, DNA extraction from whole blood was carried out using the phenol-chloroform method. The SNPs were genotyped using the polymerase chain reaction-restriction fragment length polymorphism method and validated via the direct sequencing of 20% randomly selected duplicate samples (n = 200), showing 100% concordance and confirming the accuracy of the genotyping process.

### SNP selection and construction of GRS

Three SNPs (*TCF7L2* rs7903146, *FTO* rs8050136 and rs1588413) were selected in this study based on prior established associations with metabolic traits in South Asians [17–21], as well as other populations [38–41]. Based on the assessment of genotype count, all SNPs were in Hardy-Weinberg equilibrium (*p* > 0.05) (S2 Table). An unweighted GRS was derived by summing the number of risk alleles carried by each participant, consistent with previous studies [23,42,43]. For each variant used to compute the GRS (rs7903146, rs8050136, and rs1588413), genotypes were coded as 0, 1, or 2 to represent the number of risk alleles. The total GRS was calculated by adding these values across all three SNPs. Due to the unavailable information on SNP effect sizes from large-scale GWASs in Asian Indian populations, an unweighted GRS was calculated, treating each risk allele equally. The 3-SNP GRS ranged from 0 to 5, and study participants were stratified based on the median number of risk alleles into two categories: a low-risk group (individuals with a GRS of ≤ 1 risk allele) and a high-risk group (individuals with a GRS of > 1 risk allele).

### Statistical analysis

Statistical analysis was performed using the SPSS software package (version 27; SPSS Inc., Chicago, IL, USA). Outliers were detected based on Z-scores, applying a threshold of ±3 to indicate extreme observations. The normality of variables was assessed using the Shapiro-Wilk test, and all metabolic outcomes were log-transformed prior to analysis to achieve a normal distribution. Descriptive statistics for continuous variables are reported as means and standard deviations (SDs) and as percentages for categorical variables. Differences in the mean values of continuous variables between men and women were analysed using an independent sample t-test. A comparison of categorical variables between the two groups was performed using the chi-square test. Allele frequencies were estimated using gene counting, and the goodness-of-fit chi-square test was employed to determine if the observed genotype counts were in Hardy-Weinberg equilibrium (HWE). The genotypic frequencies of selected SNPs showed no significant departure from HWE (*p* > 0.05), and the minor allele frequency for all SNPs was greater than 5% (S2 Table).

The association between the 3-SNP GRS and metabolic outcomes was examined using linear regression models adjusted for age, sex, T2D, and BMI when BMI was not an outcome. Participants were categorised into groups based on DQS values: low (<68.65), medium (68.66–87.87) and high (87.88) levels. Interactions between the GRS and DQS levels on metabolic traits were analysed using the interaction term (GRS*DQS) in the regression model, adjusting for age, gender, T2D and BMI when BMI was not an outcome. The metabolic traits examined included serum TC (mg/dL), TG (mg/dL), HDL-C (mg/dL), LDL-C (mg/dL), HbA1c (%), FPG (mg/dL), BMI (kg/m^2^) and WC (cm). For each interaction effect, we reported the p_interaction_ value, F-statistic, and partial eta-squared (η²) to describe statistical significance and effect size. Effect sizes were interpreted using established thresholds, where partial η² values of approximately 0.01, 0.06, and 0.14 indicate small, medium, and large effects, respectively [44]. Bonferroni correction was applied to control for multiple testing. The adjusted *p*-value threshold was accordingly set at < 0.0062. This was calculated based on one GRS evaluated against eight metabolic outcomes (TC, TG, HDL-C, LDL-C, HbA1c, FPG, BMI and WC) and one lifestyle factor (DQS level), resulting in eight tests.

A retrospective power calculation was not performed, given the lack of previously published effect sizes for gene–diet interaction for South Asians. However, a post hoc power analysis was conducted using G*Power 3.1 to assess the statistical power of the linear multiple regression model employed in this study. Assuming a small effect size (f² = 0.02), a significance level (α) of 0.05, a total sample size of 1020, and two predictors in the model, the computed power (1 – β) was 0.987. This high power indicates a 98.7% probability of correctly rejecting the null hypothesis, suggesting that the sample size was sufficient to detect the specified effect size.

### Ethics approval and consent to participate

Ethical approval was granted by the Madras Diabetes Research Foundation Ethics Committee, India, and all clinical procedures followed the Declaration of Helsinki and ICH-GCP guidelines. Written informed consent was obtained from all participants prior to participation. Additional information regarding the ethical, cultural, and scientific considerations specific to inclusivity in global research is included in the Supporting Information (S1 Checklist).

## Results

### Characteristics of study participants

The anthropometric, biochemical, and lifestyle characteristics of the study participants are presented in Table 1. The mean age was 44.89 ± 12.11, with no significant difference between men and women (45.47 ± 12.66 vs. 44.43 ± 11.64 years; *p* = 0.174). Women had significantly higher BMI, HC, and obesity rates (*p* < 0.001 for all), whereas men had significantly higher WC (*p* < 0.001). Significant differences were also found between the two groups in terms of diastolic blood pressure, TC, TG, HDL-C, LDL-C and VLDL-C levels (*p* < 0.05 for all). Physical activity levels were low overall, with 71% of participants classified as sedentary, and sedentary behaviour was more prevalent among women than men (76.7% vs. 64.9%; p < 0.001). Macronutrient intake, DQS and other lifestyle characteristics also differed significantly between participants in the two groups. However, there were no significant differences in glycemic parameters between men and women (*p* > 0.05 for all).

**Table 1:** Characteristics of the study population.

Characteristics of Study Participants	n	Overall Mean ± SD	Men (n = 452) Mean ± SD	Women (n = 568) Mean ± SD	*P* Value
Age (year)	1020	44.89 ± 12.11	45.47 ± 12.66	44.43 ± 11.64	0.174
BMI (kg/m2)	1019	24.50 ± 4.42	23.77 ± 4.25	25.07 ± 4.55	**<0.001** ^a^
WC (cm)	981	86.83 ± 11.43	88.35 ± 10.99	85.61 ± 11.63	**<0.001** ^a^
HC (cm)	981	95.85 ± 9.70	92.51 ± 8.24	98.51 ± 9.95	**<0.001** ^a^
Educational Qualification	674				**< 0.001**
Illiterate	89	13.2%	3.3%	21.1%	
Below SSC	364	54%	50.7%	56.7%	
Only SSC	153	22.7%	28%	18.4%	
Graduate	51	7.6%	13%	3.2%	
Postgraduate/professional	17	2.5%	5%	0.5%	
Income level	646				**< 0.001**
<RS. 2000	254	39.3%	29.9%	46.6%	
RS. 2000–5000	314	48.6%	54.4%	44.1%	
RS. 5000–10000	59	9.1%	10%	8.5%	
> RS.10000	19	3%	5.7%	0.8%	
Obesity Classification (%)	1020				**< 0.001**
Non-obese	580	56.9%	63.1%	51.9%	
Obese	440	43.1%	36.9%	48.1%	
T2D Classification (%)	1020				0.652
NGT		48.1%	47.3%	48.8%	
T2D		51.9%	52.7%	51.2%	
Hypertension	1020				0.597
No	839	82.3%	83%	81.7%	
Yes	181	17.7%	17%	18.3	
Ischemic Heart Disease	1020				0.075
No	997	97.7%	98.7%	97%	
Yes	23	2.3%	1.3%	3%	
Physical Activity Level	936				**< 0.001**
Sedentary	667	71.3%	64.9%	76.7%	
Moderate	213	22.8%	23.3%	22.3%	
Vigorous	56	6%	11.8%	1%	
HbA1c (%)	1014	7.21 ± 2.31	7.27 ± 2.35	7.15 ± 2.28	0.411^a^
FPG (mg/dl)	1018	123.95 ± 59.74	122.32 ± 56.37	125.25 ± 62.30	0.679^a^
SBP (mmhg)	1019	122.08 ± 19.97	122.46 ± 20.62	121.78 ± 19.45	0.627^a^
DBP (mmhg)	1019	75.06 ± 12.13	76.07 ± 12.32	74.26 ± 11.92	**0.021** ^a^
Serum TC (mg/dl)	1017	188.04 ± 39.67	182.46 ± 37.20	192.47 ± 41.02	**<0.001** ^a^
Serum TG (mg/dl)	1016	145.07 ± 82.95	151.58 ± 88.06	139 ± 78.35	**0.018** ^a^
HDL-C (mg/dl)	1018	42.24 ± 9.64	39.64 ± 8.98	44.31 ± 9.65	**<0.001** ^a^
LDL-C (mg/dl)	987	117.02 ± 32.36	113.03 ± 30.66	120.14 ± 33.32	**<0.001** ^a^
VLDL-C (mg/dl)	1017	28.84 ± 16.26	30.23 ± 17.31	27.73 ± 15.29	**0.025** ^a^
Total energy intake (kcal)	1020	2548.09 ± 795.70	2801.86 ± 799.56	2346.14 ± 30.75	**< 0.001**
Protein (g)	1020	71.87 ± 22.74	78.91 ± 22.94	66.28 ± 20.98	**< 0.001**
Carbohydrate (g)	1020	411.09 ± 130.68	446.35 ± 128.25	383.02 ± 125.81	**< 0.001**
Dietary Fiber (g)	1020	31.62 ± 11.41	35.04 ± 12	28.91 ± 10.15	**< 0.001**
Fat (g)	1020	66.45 ± 26.19	73.12 ± 26.92	61.14 ± 24.34	**< 0.001**
Total SFA (g)	1020	24.52 ± 10.04	26.76 ± 10.29	22.73 ± 9.47	**< 0.001**
Total MUFA (g)	1020	19.73 ± 8.27	21.71 ± 8.56	18.16 ± 7.68	**< 0.001**
Total PUFA (g)	1020	18.40 ± 9.75	20.50 ± 10.08	16.72 ± 9.14	**< 0.001**
Total TFA (g)	1020	0.18 ± 0.31	0.23 ± 0.41	0.15 ± 0.20	**< 0.001**
Pulses and legumes (g)	1020	52.01 ± 21.30	58.37 ± 22.25	46.96 ± 19.07	**< 0.001**
Milk and its products (g)	1020	403.19 ± 227.08	434.58 ± 227.90	378.20 ± 223.50	**< 0.001**
Fruits and vegetables (g)	1020	337.98 ± 163.07	372.65 ± 170.85	310.39 ± 151.17	**< 0.001**
White rice (g)	1020	339.22 ± 125.34	365.10 ± 126.60	318.62 ± 120.53	**< 0.001**
Edible fats and oils (g)	1020	32.40 ± 13.84	35.75 ± 14.17	29.73 ± 12.98	**< 0.001**
Added Sugar (g)	1020	13.25 ± 15.09	14.56 ± 16.50	12.20 ± 13.80	**0.015**
Added salt (g)	1020	8.98 ± 3.55	10.04 ± 3.60	8.15 ± 3.27	**< 0.001**
Poultry and eggs (g)	1020	25.99 ± 20.66	29.26 ± 21.71	23.14 ± 19.44	**< 0.001**
3-SNP GRS (%)	1020				
Low ≤ 1		56.6%	53.5%	59%	0.082
High > 1		43.4%	46.5%	41%	
DQS levels (%)	1020				**< 0.001**
Low	340	33.3%	64.9%	76.7%	
Medium	340	33.3%	23.3%	22.3%	
High	340	33.3%	11.8%	1%	

Data are represented in mean ± SD for continuous variables and percentages for categorical variables. ^a^The analysis was carried out using log-transformed variables. *P*-values were obtained using an independent sample t-test for continuous variables and a chi-square test for categorical variables. Values in bold represent statistical significance (*p* < 0.05).

NGT, normal glucose tolerance; T2D, type 2 diabetes; SSC, secondary school certificate, WC, waist circumference; HC, hip circumference; HbA1c, glycated haemoglobin; FPG, fasting plasma glucose; TC, total cholesterol; TG, triglycerides; HDL, high-density lipoprotein; LDL, low-density lipoprotein; VLDL, very low-density lipoprotein; SBP, systolic blood pressure, DBP, diastolic blood pressure; SFA, saturated fatty acids; MUFA, monounsaturated fatty acids; PUFA, polyunsaturated fatty acids; TFA trans fatty acid; GRS, genetic risk score; DQS, diet quality score.

### Association of GRS with metabolic disease-related traits

There were significant associations between the 3-SNP GRS and HbA1c (*p* = 0.006) and FPG (*p* = 0.005) levels after adjusting for age, sex, T2D and BMI, where individuals with > 1 risk allele had higher FPG and HbA1c levels than those with ≤ 1 risk allele (Table 2). However, no significant associations were observed between the GRS and serum TC, TG, HDL-C, LDL-C, BMI and WC (*p* > 0.05 for all).

**Table 2:** Association between 3-SNP GRS and cardiometabolic traits.

Metabolic parameters	GRS ≤ 1 N = 577	GRS > 1 n = 443	*p* Value^a^
BMI (kg/m^2^)	24.26 ± 4.42	24.80 ± 4.41	0.356
WC (cm)	85.66 ± 11.54	88.35 ± 11.11	0.250
FPG (mg/dl)	115.55 ± 55.57	134.89 ± 63.17	**0.005**
HbA1c (%)	6.85 ± 2.1	7.67 ± 2.48	**0.006**
Serum TC (mg/dl)	185.44 ± 38.48	191.43 ± 40.97	0.443
Serum TG (mg/dl)	139.71 ± 80.29	152.05 ± 85.88	0.543
HDL-C (mg/dl)	42.41 ± 9.64	42.02 ± 9.64	0.796
LDL-C (mg/dl)	114.7 ± 31.16	120.11 ± 33.68	0.190

Data are represented in mean ± SD. *P* values were obtained using a linear regression model adjusted for age, sex, T2D, and BMI when BMI was not an outcome. ^a^The analysis was carried out using log-transformed variables. Values in bold represent statistical significance (*p* < 0.05).

Abbreviations: BMI, body mass index; WC, waist circumference; FPG, fasting plasma glucose; HbA1c, glycated haemoglobin; TC, total cholesterol; TG, triglycerides; HDL, high-density lipoprotein; LDL, low-density lipoprotein.

### Interaction of GRS with DQS on metabolic disease-related traits

Significant interactions were identified between the 3-SNP GRS and DQS levels on serum TC (F = 5.72, p_interaction_ = 0.003) and LDL-C (F = 5.43, p_interaction_ = 0.005) levels after adjusting for the following confounders: age, sex, BMI and T2D. The interaction explained 1.1% of the variance in log-transformed TC and LDL-C after controlling for all other variables in the model (partial η² = 0.011 for both), indicating a small yet meaningful interaction effect. The results remained statistically significant after correction for multiple testing. Further analysis by DQS tertile showed a significant difference in serum TC levels between GRS groups at the low DQS level, where individuals with > 1 risk allele had higher TC levels than those with ≤ 1 risk allele (*p* < 0.001) but not at the medium or high DQS levels (*p* = 0.521, *p* = 0.621, respectively) (Fig 1). A similar trend was observed for LDL-C, with a significant difference at the low DQS level (*p* < 0.001), but not at medium or high DQS levels (*p* = 0.670, *p* = 0.776, respectively) (Fig 2). A significant interaction was observed between the 3-SNP GRS and DQS on HbA1c level; however, this interaction did not remain significant after correction for multiple testing. No significant interactions were observed between the GRS and DQS on other measured traits (*p* > 0.05 for all) (Table 3).

**Table 3:** Interaction between 3-SNP GRS with DQS levels on cardiometabolic traits.

	3-SNP GRS*DQS Levels							
Metabolic parameters	BMI (kg/m^2^)	WC (cm)	FPG (mg/dl)	HbA1c (%)	Serum TC (mg/dl)	Serum TG (mg/dl)	HDL-C (mg/dl)	LDL-C (mg/dl)
p_interaction_	0.159	0.867	0.282	0.019	**0.003**	0.795	0.239	**0.005**
F	1.84	0.14	1.26	3.97	5.72	0.23	1.43	5.43
Partial η²	0.004	0.0002	0.003	0.008	0.011	0.0004	0.003	0.011

P_interaction_ values were obtained using a linear regression model. F-values of the F-test. Partial η² values represent effect size estimates for each model effect.

Model was adjusted for age, sex, T2D and BMI when BMI was not an outcome.

The analysis was carried out using log-transformed variables.

Abbreviations: BMI, body mass index; WC, waist circumference; FPG, fasting plasma glucose; HbA1c, glycated haemoglobin; TC, total cholesterol; TG, triglycerides; HDL, high-density lipoprotein; LDL, low-density lipoprotein.

**Fig 1:**
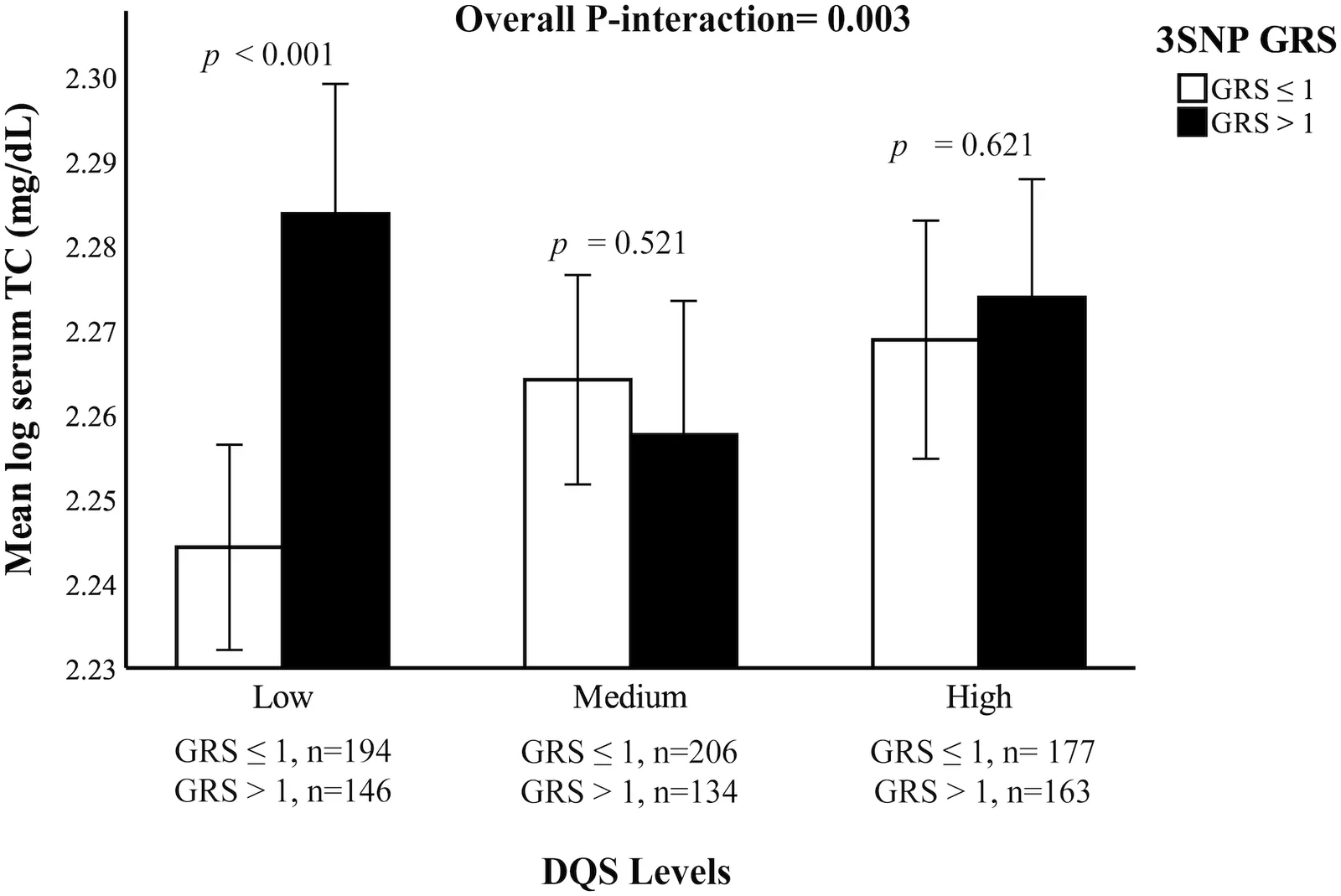
Differences in mean log-transformed serum total cholesterol (TC) levels (mg/dL) by diet quality score (DQS) levels and 3-SNP genetic risk score (GRS) groups. The DQS levels are low (DQS < 68.65), medium (DQS = 68.66–87.87) and high (DQS > 87.88). White bars represent individuals in the low GRS group (GRS ≤ 1 risk allele), and black bars represent those in the high GRS group (GRS > 1 risk allele). Error bars indicate the standard error of the mean. Significant differences in serum cholesterol levels between GRS groups were observed at the low DQS level (p < 0.001) but not at the medium (p = 0.521) or high (p = 0.621) DQS levels. The interaction between DQS levels and GRS groups was significant (p_interaction_ = 0.003).

**Fig 2:**
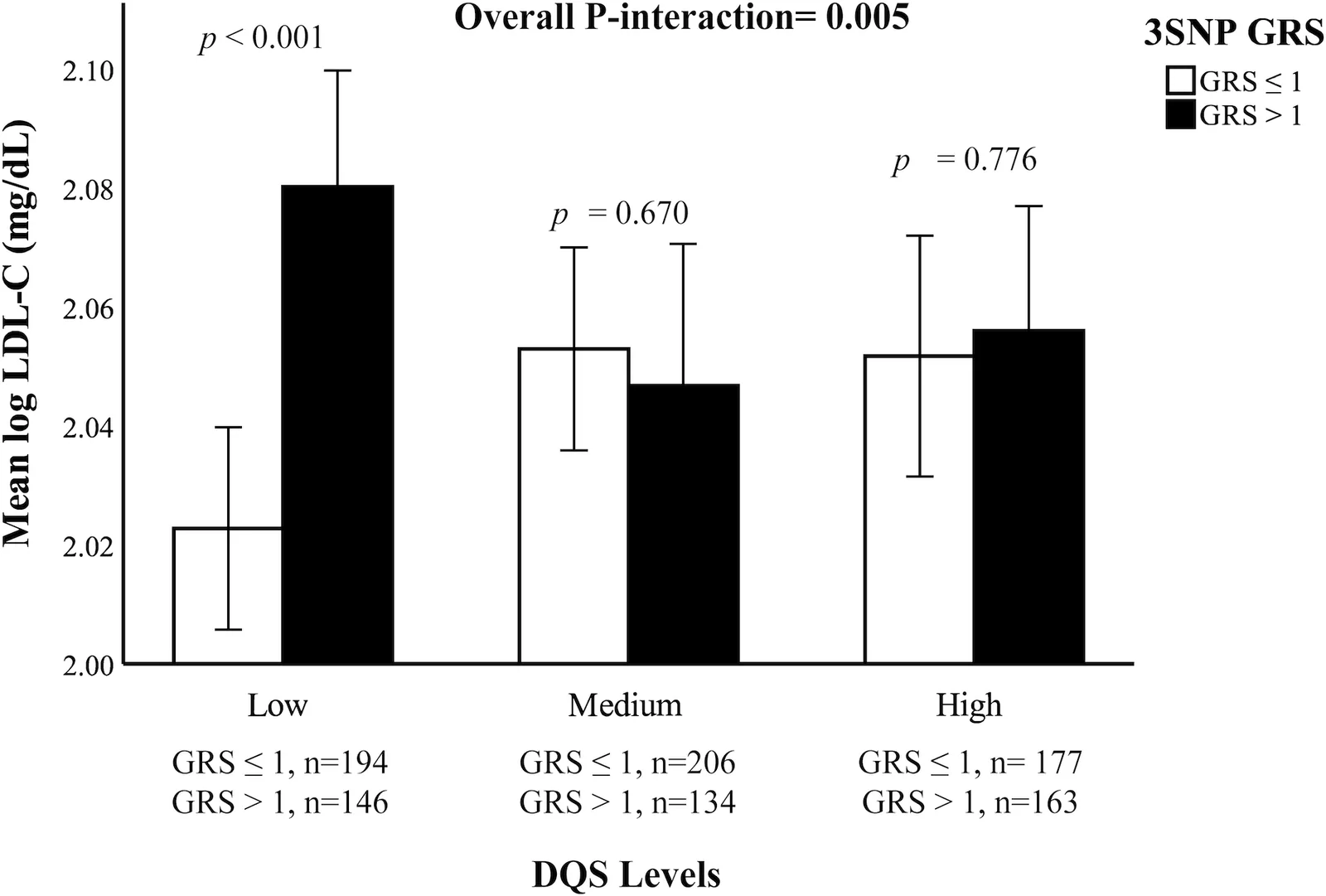
Differences in mean log-transformed LDL-C levels (mg/dL) by diet quality score (DQS) levels and 3-SNP genetic risk score (GRS) groups. The DQS levels are low (DQS < 68.65), medium (DQS = 68.66–87.87) and high (DQS > 87.88). White bars represent individuals in the low GRS group (GRS ≤ 1 risk allele), and black bars represent those in the high GRS group (GRS > 1 risk allele). Error bars indicate the standard error of the mean. Significant differences in LDL cholesterol levels between GRS groups were observed at the low DQS level (p < 0.001) but not at the medium (p = 0.670) or high (p = 0.776) DQS levels. The interaction between DQS levels and GRS groups was significant (p_interaction_ = 0.005).

## Discussion

To the best of our knowledge, this is the first study to examine the interaction between genetic risk and diet quality on multiple CMD-related outcomes in the Asian Indian population using a culturally tailored DQS. We identified significant interactions between a 3-SNP GRS and diet quality levels on lipid parameters. Individuals with a high genetic risk (GRS > 1 risk allele) had significantly increased TC and LDL-C levels when consuming a low-quality diet compared to those with a low genetic risk (GRS ≤ 1 risk allele). Although the interaction explained a small proportion of variance in lipid outcomes (partial η² ≈ 0.01), effect sizes of this magnitude are expected in multifactorial gene–diet analyses and remain meaningful in complex-trait models, with statistically robust effects observed for TC (F = 5.73, *p* = 0.003) and LDL-C (F = 5.43, *p* = 0.005) [45]. Similar modest interaction effects have been reported in nutrigenetic studies across Asian populations, where small effect sizes remain relevant when considered in the context of cumulative genetic susceptibility and dietary exposures [46,47]. Our findings suggest that among Asian Indians with elevated genetic risk, adherence to a poor-quality diet may increase susceptibility to lipid abnormalities. In line with recent evidence that emphasises the high burden of CMDs and the increasing prevalence of dyslipidemia in Asian Indians [5,8], our results highlight an essential target for dietary intervention. According to recent global estimates, dietary risks and elevated LDL-C levels account for over 50% of the global CVD burden [1]. Hence, current evidence emphasises the importance of improving diet quality to mitigate lipid abnormalities and overall CMD risk, particularly among genetically predisposed Asian Indians.

This study provided novel data from a high-risk population, offering insights that can be compared with findings from other ethnic groups. However, differences in score composition, selected genetic variants, and outcome measures limited direct cross-study comparisons. In a related study context, a 16-year follow-up of 3,152 adults from the Malmö Diet and Cancer cohort showed that poor diet quality was associated with a decrease in HDL-C levels among individuals with a low predisposition to low HDL-C, whereas no such association was observed among those with a high genetic risk (p_interaction_ = 0.04) [48]. Conversely, a large-scale study of 24,788 adults from the same cohort found no interaction between lipid-related GRS and diet quality on overall CVD risk; however, a stratified analysis revealed a significant interaction between an LDL-C-specific GRS and DQS on ischemic stroke incidence [49]. In the present study, stratified analyses showed that individuals with a high genetic risk had significantly higher lipid levels than those with a low genetic risk, but only within the low-quality diet group; no such differences were observed in the medium- or high-quality diet groups. Similar findings were reported in a study assessing the Mediterranean diet, where carriers of the *TCF7L2* rs7903146 risk genotype exhibited increased TC and LDL-C concentrations when adherence was low, while high adherence offset this effect [50]. Collectively, these findings suggested that poor diet quality may exacerbate genetic predisposition to dyslipidemia; however, the scarcity and heterogeneity of lipid-focused studies reveal substantial gaps in evidence.

In this study, the nutrigenetic approach utilised the metabolic GRS of three SNPs: *TCF7L2* rs7903146, *FTO* rs8050136, and rs1588413. Among these, *TCF7L2* rs7903146 is a well-established marker for T2D across multiple ethnic groups [40,41], including Asian Indians [19,21], and it has also been linked to lipid abnormalities [19,51–53]. *TCF7L2*, a key transcription factor in the Wnt signalling pathway, regulates β-cell function and insulin secretion and influences lipid metabolism through insulin-independent mechanisms [51,52,54]. In a North Indian case-control study involving 556 T2D cases and 537 controls, *TCF7L2* was identified as a genetic risk factor for TC levels, showing a strong BMI-independent association with plasma TC and LDL-C levels among Asian Indians [19]. Similarly, *FTO* variants have been consistently associated with T2D risk in Caucasian and Asian populations [17,18,38,40,55]. A genome-wide association study of 159,940 individuals from diverse ethnic groups, including African, South Asian, East Asian, and European participants, identified 60 genetic variants linked to HbA1c, including those within the *FTO* and *TCF7L2* genes [56]. *FTO,* while primarily associated with appetite control and energy homeostasis, may affect lipid metabolism via hepatic signalling pathways and the modulation of lipid-related gene expression [39,57,58]. The *FTO* gene has been linked to altered lipid profiles in various populations, including South Asian people [59–63]. In addition to the reported gene–diet quality interaction on lipid traits, the present study also found significant associations between the GRS and glycaemic traits, with individuals carrying more than one risk allele having significantly higher HbA1c and FPG levels than those with one or no risk alleles. These findings align with those of prior reports and underscore the important role of *FTO* and *TCF7L2* variants in influencing both glycemic control and lipid metabolism in the Asian Indian population.

The observed interaction likely reflects the integration of nutrient-sensitive pathways with genetic predisposition, initiating molecular responses that influence lipid metabolism. In South Asians, diet quality has been associated with shifts in specific lipid markers, including phosphatidylcholines, phosphatidylethanolamines, cholesterol esters, and sphingomyelins, which are functionally linked to fatty acid metabolism, lipid transport, and inflammatory processes [64,65]. These findings suggest that diet may influence lipid profiles by altering the lipidomic composition relevant to these metabolic processes. Poor dietary patterns marked by high intake of fried snacks, sweets, and high-fat dairy are also associated with elevated HOMA-IR, indicating increased insulin resistance, an early metabolic indicator of T2D [66]. Additionally, national dietary assessments have linked poor diet quality to imbalanced macronutrient ratios and inadequate essential micronutrient intake in Indian adults, further contributing to dysregulated lipid profiles [28,67]. Diets high in refined grains, red and processed meats, and full-fat dairy, as well as those low in fruits, vegetables, fish and whole grains, have been associated with increased systemic inflammation, higher lipoprotein-associated insulin resistance, and elevated levels of pro-inflammatory markers (CRP, IL-6, TNF-α, and complement C3), alongside decreased adiponectin [68]. These inflammatory responses may disturb lipid metabolism through inflammatory signalling. While the gene–diet interactions observed here are mechanistically consistent with known biological pathways, the cross-sectional design of this study limits causal inference due to the simultaneous assessment of exposures and outcomes, thereby preventing the determination of temporal sequencing between diet and metabolic traits. Consequently, changes in gene–diet interactions over time and the cumulative effects of long-term dietary exposures cannot be assessed, and the findings should therefore be interpreted as associative [69,70]. Overall, the findings suggest that poor dietary quality may amplify the impact of *FTO* and *TCF7L2* variants on lipid metabolism through nutrient-sensitive regulatory mechanisms. However, the exact mechanisms through which poor diet quality modifies gene-lipid associations remain difficult to isolate, likely due to the combined influence of multiple, overlapping dietary exposures. This highlights the need for further mechanistic research in culturally specific contexts.

Diet quality scores are increasingly used in epidemiological research as composite measures of adherence to dietary guidelines, providing a structured approach to evaluate overall dietary patterns and assess dietary risk factors for chronic diseases [71,72]. Interest in these scores has grown partly due to the limitations of single-nutrient approaches, which often fail to capture the complexity of the overall diet and the interactive nature of nutrients [25,73]. Furthermore, dietary scores reduce the risk of chance findings from multiple testing and improve the ability to detect associations when the impact of individual nutrients is modest [73]. As such, they offer a more integrated and informative approach for evaluating the cumulative effect of dietary components on metabolic outcomes in population-based studies [74,75]. A well-documented example comes from a Swedish study of 17,126 participants recruited from the Malmö Diet and Cancer cohort, where a high-quality diet was associated with a reduced risk of CVD events compared to a low-quality diet [76]. Notably, the overall DQS showed a stronger association with disease risk than any of its components individually. Likewise, in a gene–diet interaction analysis of 7018 Spanish adults, significant interactions between the *TCF7L2* rs7903146 variant and adherence to the Mediterranean diet were observed for fasting glucose and lipid levels, whereas no such interactions were found with individual dietary variables [77]. This highlights the synergistic effect of the multiple dietary components acting together and the advantage of using a composite dietary score for assessing the diet–disease relationship.

Several diet quality indices have been developed in Western populations [78–80]. However, these indices may not fully capture the dietary patterns and metabolic responses specific to Asian Indians, which are influenced by both culture and genetics. India is undergoing a significant transition in terms of both the quality and quantity of diets, characterised by higher consumption of refined grains, such as white rice and refined wheat; insufficient intake of high-quality protein, fibre, fruits, and vegetables, and reduced dietary diversity [12,81]. An assessment of national dietary intake reveals that carbohydrates account for 64% of total energy intake, followed by fat (24%) and protein (12%), with refined cereals contributing substantially to energy intake [82]. Among affluent Indian adults, 78% had a poor-quality diet [28]. Considering these shifts, the IDQS used in our study provides a culturally appropriate tool, tailored for capturing dietary factors relevant to metabolic disease risk in the Indian population through eight food groups [27], several of which have been linked to lipid outcomes and overall CMD risk [83–86]. The alignment between the DQS components and known metabolic risk factors supports the relevance of our findings within this population.

Our study has several strengths. Firstly, dietary data were collected using a validated FFQ, and diet quality was assessed with a culturally adapted score that reflects dietary patterns relevant to metabolic risk in Asian Indians. Secondly, a comparatively large sample size was drawn from a distinct ethnic group with limited representation in gene-diet interaction research. Thirdly, the use of a representative cohort from Chennai supports the potential generalizability of the findings to broader South Asian populations. Fourthly, analyses were corrected for multiple testing and adjusted for relevant confounders to strengthen the robustness of the findings. Another strength was using a combined GRS derived from well-established variants, which offered greater predictive power than SNP-based analyses [24]. Additionally, a minor modification was applied to the IDQS, which included a weighting approach to the scores of protective and harmful food groups, as well as excluding the cooking oil type component. These adjustments were informed by the current data and are generally recommended to prevent score overestimation and redundancy [87]. However, certain limitations should be acknowledged— although DQSs offer a practical measure of dietary patterns, they rely on self-reported data, which may introduce recall bias and misreporting [71], potentially affecting the precision of capturing overall diet quality. Accordingly, future studies would benefit from incorporating more objective dietary assessment methods, such as repeated 24-hour dietary recalls or food diaries, to improve the accuracy of dietary intake estimation. Moreover, the score used does not account for additional food variables and contextual factors, such as food preparation methods, dietary diversity, or evolving consumption trends, particularly the increasing intake of processed and fast foods. The cross-sectional design also limits the ability to derive causal inference due to the simultaneous measurement of exposures and outcomes [69]. Finally, this study offers valuable insights into how evidence from gene–diet interaction research can inform more tailored dietary recommendations by identifying population subgroups that may benefit most from higher-quality dietary patterns. Integrating such findings from diverse populations into public health policies and precision nutrition strategies may further strengthen the development of targeted context-specific interventions [88]. Taken together, these results support more focused, prevention-oriented dietary approaches for individuals at higher CMD risk while remaining feasible at the population level.

## Conclusion

In summary, this study provides novel evidence that diet quality, assessed using a culturally relevant dietary index, modifies the impact of genetic risk and lipid profile in the Asian Indian population. Significant interactions were observed between a metabolic GRS and diet quality, influencing TC and LDL-C levels. Specifically, individuals with high genetic risk exhibited greater abnormalities in lipid levels when adhering to a low-quality diet than those with low genetic risk. The findings emphasise the potential of dietary interventions for attenuating genetic susceptibility to dyslipidemia and highlight the value of integrating both dietary and genetic data into personalised cardiometabolic risk management strategies. Future research should aim to replicate these findings in longitudinal and prospective cohort studies using comparable methods to track dietary changes over time and examine how GRS–diet quality interactions may help predict incident dyslipidemia and CMD. Expanding the genetic framework to include additional lipid-related variants may further refine risk stratification. In addition, well-designed dietary intervention trials are needed to assess the effects of targeted dietary modifications and specific DQS components (e.g., refined grains, edible fats, added sugar) on genetic susceptibility and lipid metabolism. Incorporating oxidative stress and antioxidant markers may also help clarify the biological pathways linking diet, genetic risk, and cardiometabolic outcomes.

## Supporting information

S1 Table Descriptive Statistics for Quintiles of Food Groups. (DOCX)

S2 Table Allele frequencies and Hardy-Weinberg Equilibrium p-values. SNP, single-nucleotide polymorphism; *TCF7L2*, transcription factor 7-like2; *FTO*, Fat mass and obesity associated.
(DOCX)

S1 Checklist Inclusivity in global research questionnaire. (DOCX)
