# Supplementary material for: Low diet quality increases the genetic risk of high blood cholesterol levels in Asian Indian adults [CURES – 165]
Source: PLoS One. 2026 Oct 14;21(10):e0357182. doi: 10.1371/journal.pone.0357182 (PMC13667832; doi:10.1371/journal.pone.0357182)
Supplement: Supplementary file 1 [file pone.0357182.s001.docx]

|  | **Mean ± SD** | | |
| --- | --- | --- | --- |
| **Food groups (g/day)** | **Low intake**  **(Q1)** | **Medium Intake**  **(Q2-Q4)** | **High Intake**  **(Q5)** |
| Pulses and legumes | 32.12 ± 10.43 | 48.88 ± 14.05 | 81.31 ± 16.91 |
| Milk and its products | 167.19 ± 89.56 | 365.51 ± 127.22 | 752.21 ± 139.50 |
| Fruits and vegetables | 216.56 ± 69.49 | 30510 ± 109.16 | 558.02 ± 161.42 |
| White rice | 248.06 ± 74.87 | 327.34 ± 105.87 | 466.01 ± 120.78 |
| Edible fats and oils | 22.13 ± 7.38 | 30.73 ± 10.82 | 47.67 ± 14.38 |
| Added Sugar | 2.92 ± 3.26 | 8.34 ± 6.66 | 38.37 ± 13.71 |
| Added salt | 6.50 ± 2.07 | 8.37 ± 2.58 | 13.31 ± 3.55 |
| Poultry and eggs | 8.40 ± 8.05 | 21.56 ± 11.85 | 56.91 ± 18.08 |
