# Supplementary material for: Low diet quality increases the genetic risk of high blood cholesterol levels in Asian Indian adults [CURES – 165]
Source: PLoS One. 2026 Oct 14;21(10):e0357182. doi: 10.1371/journal.pone.0357182 (PMC13667832; doi:10.1371/journal.pone.0357182)
Supplement: Supplementary file 2 [file pone.0357182.s002.docx]

| **SNP rs** | **Gene** | **Chromosomal Location**  **(GRCh38)** | **SNP**  **Location** | **Genotype Count** | **Allele Freq**  **(%)** | **Alleles**  **Risk>other** | **MAF** | **HWE**  **P-value** |
| --- | --- | --- | --- | --- | --- | --- | --- | --- |
| rs7903146 | *TCF7L2* | 10:112998590 | Intron variant | CC= 487  CT= 422  TT= 111 | C= 68  T= 32 | C>T | 0.32 | 0.175 |
| rs8050136 | *FTO* | 16:53782363 | Intron variant | CC= 829  CA= 178  AA= 13 | C= 90  A= 10 | C>A | 0.1 | 0.32 |
| rs1588413 | *FTO* | 16:54115620 | 3 Prime UTR Variant | CC= 507  CT= 438  TT= 75 | C= 71  T= 29 | C>T | 0.29 | 0.137 |
